# Misfit accommodation mechanism at the heterointerface between diamond and cubic boron nitride

**DOI:** 10.1038/ncomms7327

**Published:** 2015-02-17

**Authors:** Chunlin Chen, Zhongchang Wang, Takeharu Kato, Naoya Shibata, Takashi Taniguchi, Yuichi Ikuhara

**Affiliations:** 1 Advanced Institute for Materials Research, Tohoku University, 2-1-1 Katahira, Aoba-ku, Sendai 980-8577, Japan; 2 Nanostructures Research Laboratory, Japan Fine Ceramics Center, 2-4-1 Mutsuno, Atsuta, Nagoya 456-8587, Japan; 3 Institute of Engineering Innovation, University of Tokyo, 2-11-16 Yayoi, Bunkyo-ku, Tokyo 113-8656, Japan; 4 National Institute for Materials Science, Tsukuba, Ibaraki 305-0044, Japan

## Abstract

Diamond and cubic boron nitride (c-BN) are the top two hardest materials on the Earth. Clarifying how the two seemingly incompressible materials can actually join represents one of the most challenging issues in materials science. Here we apply the temperature gradient method to grow the c-BN single crystals on diamond and report a successful epitaxial growth. By transmission electron microscopy, we reveal a novel misfit accommodation mechanism for a {111} diamond/c-BN heterointerface, that is, lattice misfit can be accommodated by continuous stacking fault networks, which are connected by periodically arranged hexagonal dislocation loops. The loops are found to comprise six 60° Shockley partial dislocations. Atomically, the carbon in diamond bonds directly to boron in c-BN at the interface, which electronically induces a two-dimensional electron gas and a quasi-1D electrical conductivity. Our findings point to the existence of a novel misfit accommodation mechanism associated with the superhard materials.

## Abstract (web summary)

Interfaces between two materials often show interesting properties. Here, the authors demonstrate that diamond and cubic boron nitride, the hardest materials known, can be grown on top of each other through a novel misfit accommodation mechanism, forming a two-dimensional electron gas at the interface.

## Graphical abstract

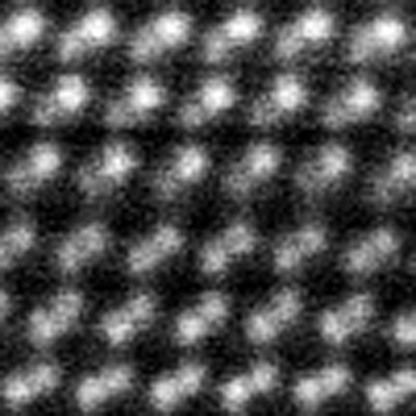

Diamond and cubic boron nitride (c-BN) attract continuing interest for years because of their superhardness originating from their extremely strong covalent bonding between the atoms with a sp^3^ configuration^1^,^2^. For decades, extensive effort has been devoted to the fabrication of these two materials with high quality and also to the discovery of their new properties for many applications^3^,^4^,^5^,^6^,^7^,^8^. However, most of the previous works have focused on each individual. It remains unclear how the two superhard materials can actually join at interface and how the lattice misfit can be accommodated due to their rigid lattices. Understanding these issues is fundamentally important for combining these two materials in order to enhance properties for many electronic and mechanical applications.

Heteroepitaxial growth is of considerable significance in both fundamental science and technological applications such as electronic, photonic and magnetic devices^9^,^10^,^11^,^12^,^13^. In general, to relieve strains, misfit dislocations are created at the interface between two materials of different lattice constants^14^. The interfacial misfit dislocations and their associated impacts on properties of materials have hitherto attracted extensive interests^15^,^16^,^17^,^18^,^19^,^20^,^21^,^22^,^23^,^24^, especially in those systems where crystal lattices are comparatively less hard to be deformed as compared with the diamond and c-BN, such as the interfaces between metals^15^,^16^,^17^, semiconductors^18^,^19^,^20^,^21^,^22^,^23^,^24^ and their combinations. However, the general knowledge on the bonding of two superhard materials with extremely rigid lattices (that is, diamond and c-BN) remains far from being developed due to the extreme difficulty in preparing one superhard material on the other^25^,^26^,^27^,^28^,^29^. Here, we use a temperature gradient method to grow a c-BN single crystal on seed crystals of diamond at high temperature and high pressure and report a successful preparation of an epitaxial c-BN/diamond heterojunction^28^. By combining advanced transmission electron microscopy (TEM) with first-principles calculations, we reveal a novel misfit dislocation mechanism at the interface and show the atomic structure and electronic bonding of the interface.

## Results

### Microstructure of the heterointerface

**Figure 1:**
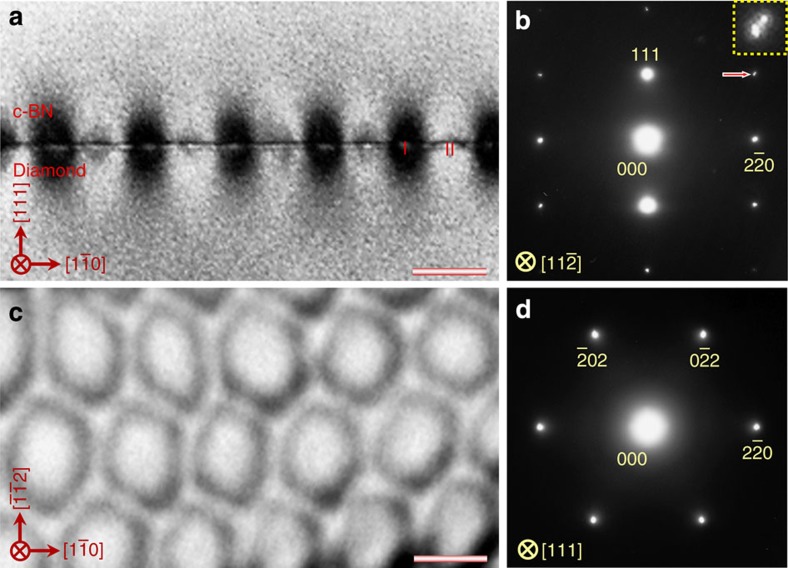
Microstructure of the (111) diamond/c-BN heterointerface. (**a**,**b**) A bright-field TEM image (**a**) and corresponding SAED pattern (**b**) taken along the 
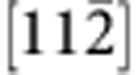
 zone axis. The cross-sectional TEM sample was used. There appears a periodic image contrast of the misfit dislocations at the edge-on interface. (**c**,**d**) Bright-field TEM image (**c**) and SAED pattern (**d**) viewed from the [111] zone axis. The misfit dislocation network consists of the periodically arranged dislocation hexagons. Scale bar, 15 nm.

Figure 1 presents bright-field TEM images and selected area electron diffraction (SAED) patterns of the c-BN/diamond heterointerface from two orthogonal directions. The images are taken at edge-on condition. There appear two sets of dark regions at the junction (marked with I and II in Fig. 1a), which are alternately aligned by an interval of ~18.4 nm, implying a semi-coherency for the interface. The dark regions have a length of ~5 nm. Clearly, there is no precipitate or secondary phase at the boundary between the c-BN and diamond. From the SAED pattern (Fig. 1b), one can identify that the interface is on (111) crystal plane as there emerge the (111) diffraction spots along the interface normal. The diffraction spots almost overlap, implying that the c-BN grows epitaxially on diamond. Moreover, there also appears a splitting of diffraction spots (Fig. 1b, inset), which can be attributed to the lattice misfit between diamond and c-BN (~1.4%).

To investigate configuration of the misfit dislocations at interface, we prepared plan-view TEM specimens with the focused ion-beam (FIB) technique. Figure 1c shows a bright-field TEM image viewed from the [111] zone axis. The misfit dislocation networks are found to be composed of hexagonal dislocation loops, which are periodically arranged by an interval of ~18.4 nm. Each loop consists of three pairs of misfit dislocations, which are along 
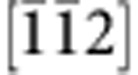
, 
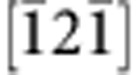
 and 
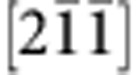
 directions. The mean interval between two adjacent loop edges is estimated to be ~5 nm, which is almost the same as length of dark regions (Fig. 1a). This indicates that the dark regions are composed of two partial dislocations that are connected by a stacking fault. The stacking fault is formed by dissociating a full 
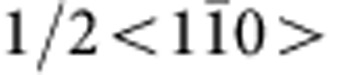
 dislocation. Figure 1d shows a SAED pattern along [111] direction, which reveals that the diffraction spots of c-BN match well to those of diamond, confirming the heteroepitaxy at the interface.

**Figure 2:**
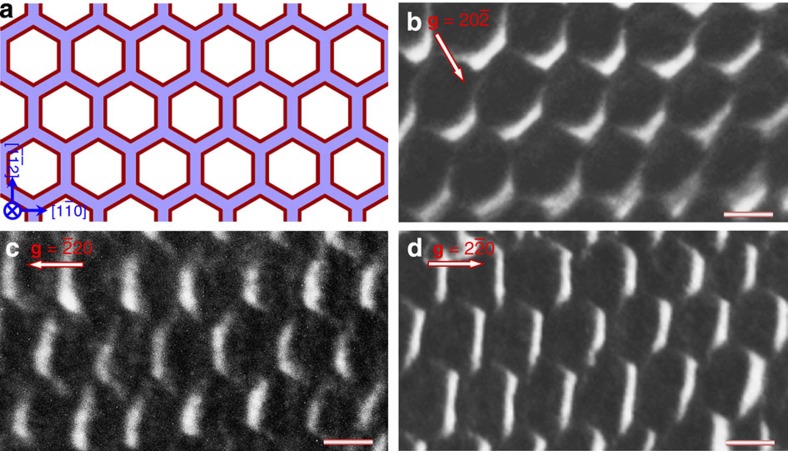
Misfit dislocations at the heterointerface. (**a**) A schematic illustration showing the misfit dislocation hexagonal loops (red lines) and the stacking faults (purple area). (**b**–**d**) Weak-beam dark-field TEM images recorded at 
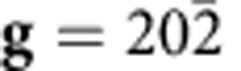
 (**b**), 
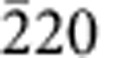
 (**c**) and 
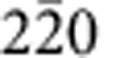
 (**d**). Among the six edges of a hexagonal unit, the two edges perpendicular to the **g** vector show a much brighter image contrast than the rest four edges that are not perpendicular to **g**. Scale bar, 15 nm.

Assuming a rigid model for the misfit dislocations, the network at the (111) interface can be composed of hexagonal units of full edge dislocations, which have a Burgers vector of 
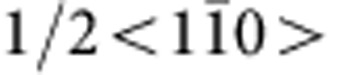
 and a dislocation line of 
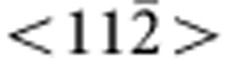
 (Supplementary Fig. 1). It is known that the 
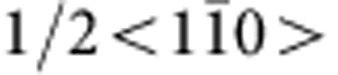
 edge dislocations in c-BN and diamond exhibit a high energy and can readily be dissociated to two 60° 
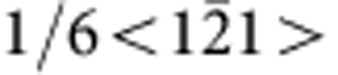
 Shockley partial dislocations, which are connected by a stacking fault^30^,^31^. We confirm that each 
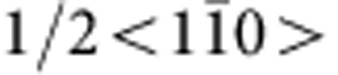
 misfit dislocation at interface undergoes a similar dissociation (Supplementary Fig. 2). Consequently, six Shockley partial dislocations form a hexagonal dislocation loop, and these periodically arranged loops are connected by a continuous stacking fault network at interface. By comparing strain energy of the 
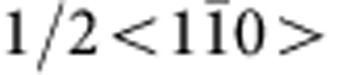
 perfect dislocation with sum of the energies of the two Shockley partial dislocations and the stacking fault (Supplementary Discussion), the dissociation is found to be energetically preferred. Figure 2a shows a sketch of the periodic misfit dislocation loops and the stacking faults on the (111) interface, which is constructed based upon the TEM image (Fig. 1c). The presence of periodic loops and continuous network can also be confirmed from the scanning TEM (STEM) image (Supplementary Fig. 3). These indicate a new misfit accommodation mechanism, that is, the lattice misfit can be accommodated by periodically arranged hexagonal dislocation loops that are connected by a continuous stacking fault network. This mechanism differs from those observed previously at the {111} interfaces between metals^15^,^16^,^17^, Si and Ge^19^,^20^ and other III-V semiconductors^21^, which reveal either no continuous stacking fault network at the interface or the accommodation of lattice misfit by misfit dislocations alone.

To determine Burgers vector of the misfit dislocations, we conducted a **g·b** analysis using the weak-beam dark-field TEM technique, as shown in Fig. 2b–d. The images were taken at 
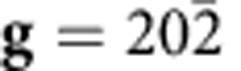
, 
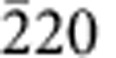
 and 
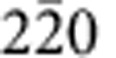
 in the region shown in Fig. 1c. The condition **g·b**=0 is defined as the extinction condition of dislocation contrast.^32^ By focusing on the image contrast of the six edges of each hexagonal unit, the two edges that are perpendicular to the **g** vector show a much brighter image contrast than the rest four edges that deviate from the **g** normal. This can be explained by the fact that the perpendicular edges have two visible Shockley partial dislocations, whereas the rest four edges have one visible and one invisible Shockley partial dislocation. Further analyses of the misfit dislocation model and dark-field images identify the Burgers vectors of the misfit dislocations as 
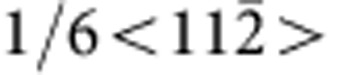
, the shortest translation vectors along 
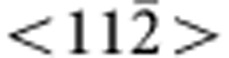
 direction. The misfit dislocations are 60° Shockley partial dislocations.

### Atomic-scale imaging of the heterointerface

**Figure 3:**
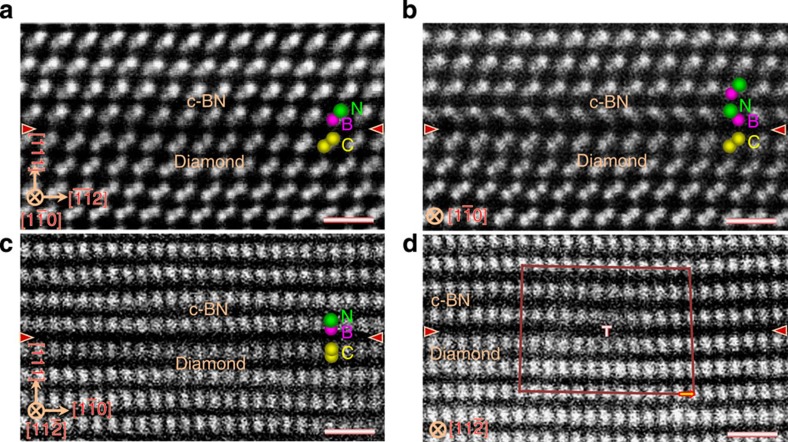
Atomic-scale structure of the interface. (**a**,**b**) HAADF STEM images taken along 
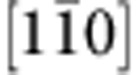
 zone axis in the directly bonded coherent area (**a**) and the stacking fault area (**b**). The interface is bonded by B and C atoms, and the stacking fault appears on the c-BN side. (**c**,**d**) HAADF STEM images taken along 
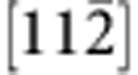
 zone axis in the coherent region (**c**) and the area containing Shockley partial dislocations (**d**). The projected Burger vector is identified as 
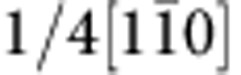
. The interface is indicated by horizontal arrows. Scale bar, 5 Å.

To offer atomic details, we performed C_s_-corrected STEM imaging of the interface from two orthogonal projections. Figure 3a shows a high-angle annular dark-field (HAADF) STEM image of the coherent area viewed from 
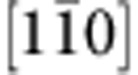
 direction. One can note that the interface is epitaxial and atomically abrupt. As the intensity of an atomic column in the HAADF imaging mode is directly proportional to *Z*^1.7^ (*Z*: atomic number)^33^, the image contrast is brighter for heavier atoms. By interpreting the image contrast, we find that C is bonded directly to B at the interface. Figure 3b shows a HAADF STEM image of a stacking fault area, from which the atomically abrupt junction (indicated by red arrows) and the direct B–C bonding can be confirmed. The stacking fault is found to locate on the c-BN side, which is attributed to the fact that the c-BN has a lower hardness and stacking fault energy than the diamond^32^,^33^. We also obtain a bright-field TEM image and a SAED pattern viewed from the 
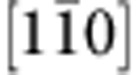
 direction, and find that misfit dislocations are inclined so that it is difficult to observe their core structures from this direction (Supplementary Fig. 4).

Figure 3c shows a HAADF STEM image of the interface from orthogonal 
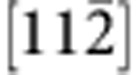
 direction. The elongated bright spot in c-BN represents two neighbouring B and N atomic columns, and that in diamond represents two neighbouring C atomic columns. Figure 3d shows a HAADF STEM image of a misfit Shockley partial dislocation taken from the 
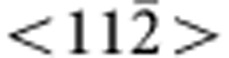
 zone axis. This axis is the line direction of misfit dislocations, thus allowing us to obtain an atomic structure of the dislocation. An extra atomic plane appears on the diamond side, which is ascribed to the fact that diamond has a smaller crystal lattice than c-BN. We further draw a Burgers circuit surrounding the dislocation and determine the projected Burger vector as 
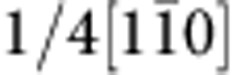
, consistent with the above observation that misfit dislocations are 60° 
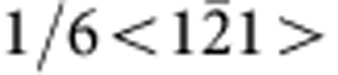
 Shockley partial dislocations. In addition, we also conducted an annular bright-field (ABF) STEM imaging, which reveals that the two adjacent Shockley partial dislocations are connected by stacking faults by an interval of ~5 nm (Supplementary Fig. 5). However, the displacement vector of the stacking fault cannot be identified in the high-resolution images taken from the 
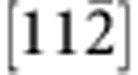
 zone axis. To render atomic structure of the {111} interface clearer, we also carry out low-pass filtering of the HAADF STEM images using fast Fourier transform, as shown in Supplementary Fig. 6.

## Discussion

**Figure 4:**
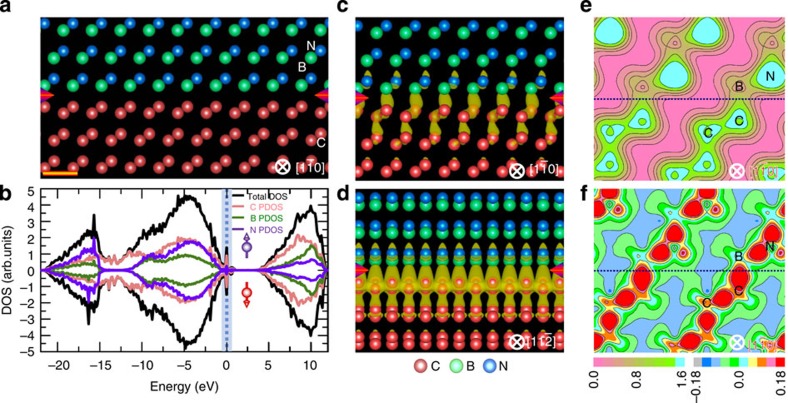
Atomic and electronic structure of the heterointerface. (**a**) Relaxed atomic model for the interface between B-terminated BN and diamond. The interface is indicated by arrows. (**b**) Total density of states (TDOS) and partial density of states (PDOS) plots of the C, B and N atom contributions for the relaxed interface. (**c**,**d**) Electron density at *E*_F_ viewed from 
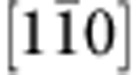
 (**c**) and 
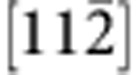
 (**d**) direction. Charges are found to be confined at the interface. (**e**,**f**) Contour plots of charge density (**e**) and charge density difference (**f**) viewed along 
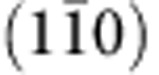
 plane. The charge density difference shows charge redistributions in the interface relative to its isolated system. The interface is marked by a horizontal line. The scale on the left correspond to charge magnitude and the one on the right correspond to charge density difference. Scale bar, 3 Å.

To gain insights into electronic structure of the interface and offer a deeper understanding of the images, we performed density functional theory calculations, taking into account both the B and N terminations for the BN. Adhesion energy calculations reveal that the B-terminated interface (Fig. 4a) has a much stronger adhesion than the N-terminated one (5.577 J m^−2^ for the B termination and 2.906 J m^−2^ for the N termination), consistent with the HAADF observations (Fig. 3a). To further verify this model, we simulated HAADF images using the determined stable interface (Fig. 4a,f) and compared them (Supplementary Fig. 7) with the experimental images (Fig. 3a,c), finding a good agreement for the both projections.

Interestingly, calculations of electronic structure predict that there emerge electronic states at Fermi level (*E*_F_) for both spins, indicating that the interface shows a full metallic character (Fig. 4b). Unexpectedly, the *E*_F_ lies precisely at a peak position of a band of electronic states, implying that the interface may be superconducting. The electronic states at *E*_F_ come mainly from the interfacial C sp orbitals and are confined to the interface, forming two-dimensional (2D) electron gas^34^,^35^,^36^. The 2D electron gas may be ascribed to the polar discontinuity across interface and may be formed on other orientations. Figure 4c,d shows spatial distribution in electronic wavefunction along 
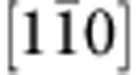
 and 
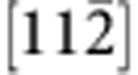
 projection. One can note that electrons are spatially separated along 
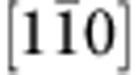
 direction, yet connected along 
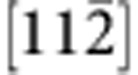
 direction via the hybridization of C sp orbitals, implying that this interface shows a quasi-1D nature in electrical conductivity. Figure 4e,f shows contour plots of charge density and density difference along 
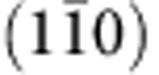
 plane. The plane was selected because it slices through C–C pairs in diamond, B–N pairs in BN and the interfacial B and C, thereby allowing us to extract maximum bonding information on this interface. Most of charges are localized on C and N atoms, and the charge distribution on each atom is severely distorted towards their neighbours (Fig. 4e), indicating that the interfacial bonds have a strong covalent character. Moreover, a large number of charges are accumulated along the interfacial B–C bonds and these charges are somewhat more than those along the C–C and B–N bonds in the respective bulks (Fig. 4f), offering an explanation to the strengthening of the interface.

Unveiling misfit accommodation mechanism and atomistic structure of the heterojunction between two superhard materials represents a significant step forward in both fundamental materials science and technological applications. We demonstrate that misfits at the interface between superhard materials could actually be accommodated by a continuous stacking fault network that is connected by periodically arranged hexagonal dislocation loops comprising six 60° Shockley partials. Because of the extreme challenge in handling superhard materials, this new mechanism offers a different view of how two inherently incompressible matters can in practice compromise. Atomically, we identify a direct B–C bonding at the interface, which triggers electronic states that have never been realized in either of the bulk materials alone. The new electronic states could be manipulated for advanced electronic device applications.

## Methods

### Sample preparation and microscopic observations

High-quality epitaxial c-BN/diamond heterojunction was prepared by growing c-BN single crystals on seed crystals of diamond under a static high pressure of 5.5 GPa at 1,600–1,700 °C (ref. 28). The temperature gradient method was applied and lithium boron nitride was used as solvent. A real picture of the as-prepared diamond/c-BN heterojunction was given in Supplementary Fig. 8. One can see that the c-BN single crystal has a size of ~0.5 mm. Thin-foil specimens for TEM and STEM imaging were prepared by FIB technique using the Hitachi FB2200 FIB system. After the FIB, the TEM specimens were cleaned by Ar ion-beam thinning with an accelerating gun voltage of 0.5–1.0 kV to reduce electron radiation damage. SAED patterns and TEM images were taken at 200 kV using the JEM-2010F (JEOL Co. Ltd) microscope with an aberration coefficient of objective lens of 1.0 mm. The diameters of the condenser aperture, objective aperture used for bright-field imaging and selected area diffraction aperture were adopted as 30, 70 and 20 μm, respectively. HAADF and ABF images were taken using 200-kV STEM (ARM200FC, JEOL) equipped with a probe corrector (CEOS, Gmbh), which offers an unprecedented opportunity to probe structures with sub-Angström resolution. For the STEM imaging, we adopted a probe size of ~1 Å and a probe convergence angle of ~25 mard. The HAADF STEM images were taken by an annular dark-field detector with a collection semiangle of 68−280 mrad. The ABF STEM images were taken by an ABF detector with a collection semiangle of 12−24 mrad.

### Image simulation

HAADF STEM image simulations were conducted using the WinHREM package (HREM Res. Inc.), which was based on the multislice method^37^. For the multislice method, samples were first divided into a number of thin slices normal to incident electron beam, and the contribution to the cross-section at every slice was then calculated. A series of sample thicknesses could be simulated by overlapping a different number of sub-slices. For HAADF image simulation, the acceleration voltage, Cs, defocus value, probe convergence angle and collection semiangle were adopted as 200 kV, 0.02 mm, 30 Å, 30 mrad and 90–170 mrad, respectively. An orthorhombic supercell with a dimension of 2.531 × 4.383 × 45.228 Å^3^ was used for the image simulations. The slice thickness and sample thickness used to simulate the image shown in Supplementary Fig. 7a were adopted as 0.633 Å and 5.064 nm, respectively, and those used to simulate the image shown in Supplementary Fig. 7b were adopted as 1.096 Å and 4.38 nm, respectively. The pixel size for the image display was set to be 0.0833 Å.

### Calculational methodology

Calculations were conducted using Vienna *ab initio* simulation package (VASP) within the framework of density functional theory^38^. We applied the projector-augmented wave method with 8 × 6 × 1 *k*-point grids and a cutoff energy of 400 eV for the interface. The generalized gradient approximation of Perdew *et al*. (PW91) was employed to address the exchange-correlation functional. Interface models consisted of a 17-layer c-BN (111) slab connected to a diamond (111) slab of at least 17 layers. A vacuum region of 10 Å was introduced to minimize the coupling along the interface normal. We applied a 1 × 1 periodic supercell along the interface plane and further quadrupled the supercell to examine the size effects. We found no remarkable influence by the supercell size. To form coherent interfaces, in-plane lattice constants of c-BN were compressed by 1.01% to match those of the harder diamond. All the atoms were fully optimized until the magnitude of force on every atom fell below 0.05 eV Å^−1^. We assessed the accuracy of the calculational methods by conducting bulk simulations. It was known that diamond belongs to *Fd-3m* space group with *a*=3.567 Å and c-BN belongs to *F-43m* space group with *a*=3.616 Å (refs 39). Bulk properties were calculated with a cutoff energy of 400 eV and 12 × 12 × 12 *k* points. The calculated optimum lattice constant of the diamond bulk is *a*=3.579 Å, 100.35% of the experimental value^39^, whereas that of the c-BN bulk is *a*=3.625 Å, 100.25% of the experimental value^40^.

## Author contributions

C.C. conducted the experiments and wrote the paper. Z.W. carried out the calculations and wrote the paper. T.K. and N.S. helped perform the microscopic imaging. T.T. and Y.I. discussed the results and directed the entire study. All authors read and commented on the paper.

## Additional information

**How to cite this article:** Chen, C. *et al*. Misfit accommodation mechanism at the heterointerface between diamond and cubic boron nitride. *Nat. Commun.* 6:6327 doi: 10.1038/ncomms7327 (2015).

## Supplementary Material

Supplementary Information Supplementary Figures 1-8, Supplementary Discussion and Supplementary References
