# Supplementary material for: Misfit accommodation mechanism at the heterointerface between diamond and cubic boron nitride
Source: Nat Commun. 2015 Feb 17;6:6327. doi: 10.1038/ncomms7327 (PMC4339885; doi:10.1038/ncomms7327)
Supplement: Supplementary Information — Supplementary Figures 1-8, Supplementary Discussion and Supplementary References [file ncomms7327-s1.pdf]

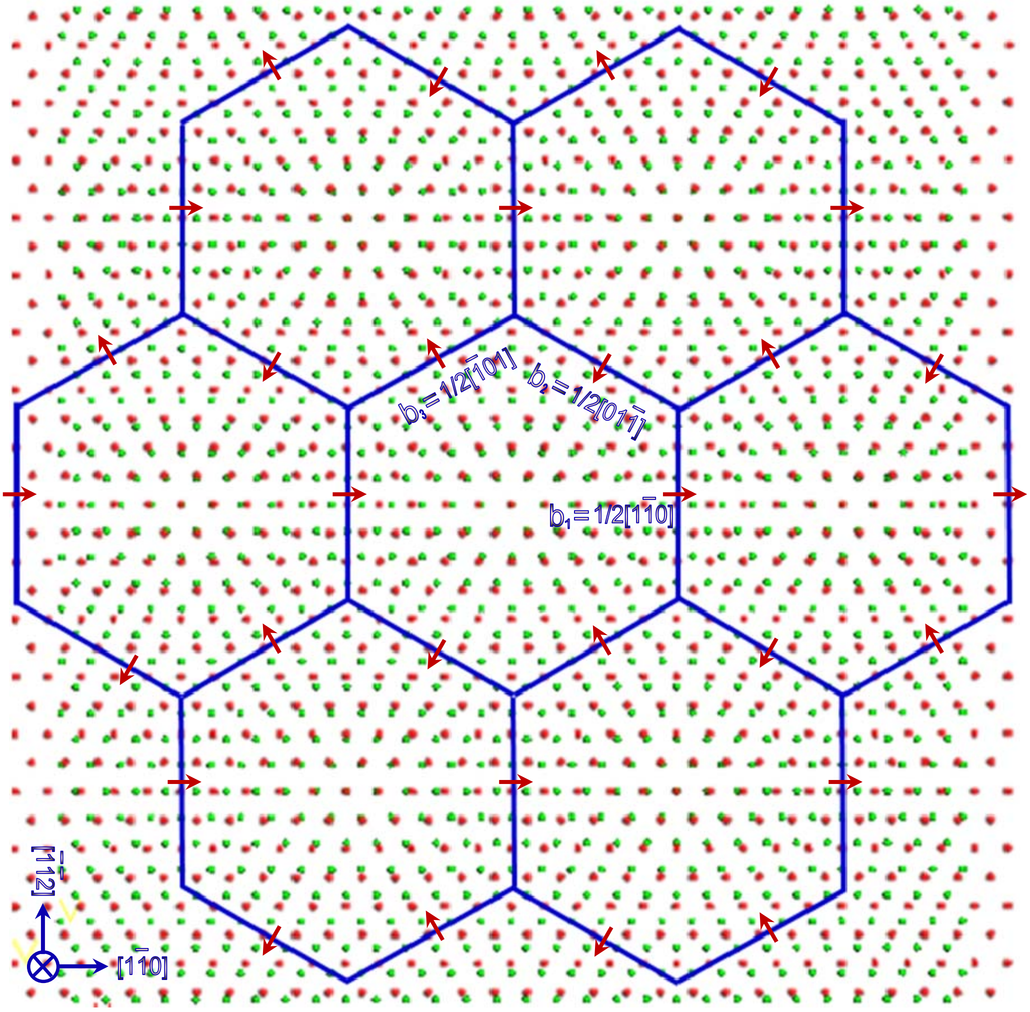

Supplementary Figure 1 | Rigid model describing the formation of misfit dislocations. A rigid model illustrating the formation of misfit dislocations at the (111) diamond/c-BN interface. The red and green lattices denote the c-BN and the diamond lattices, respectively. The viewing direction is along the [111] zone axis. The misfit dislocation network consists of hexagonal units with full edge dislocations. The edge dislocations have Burgers vectors of $1/2\langle 1\bar{1}0\rangle$ and a dislocation line of $\langle 11\bar{2}\rangle$.

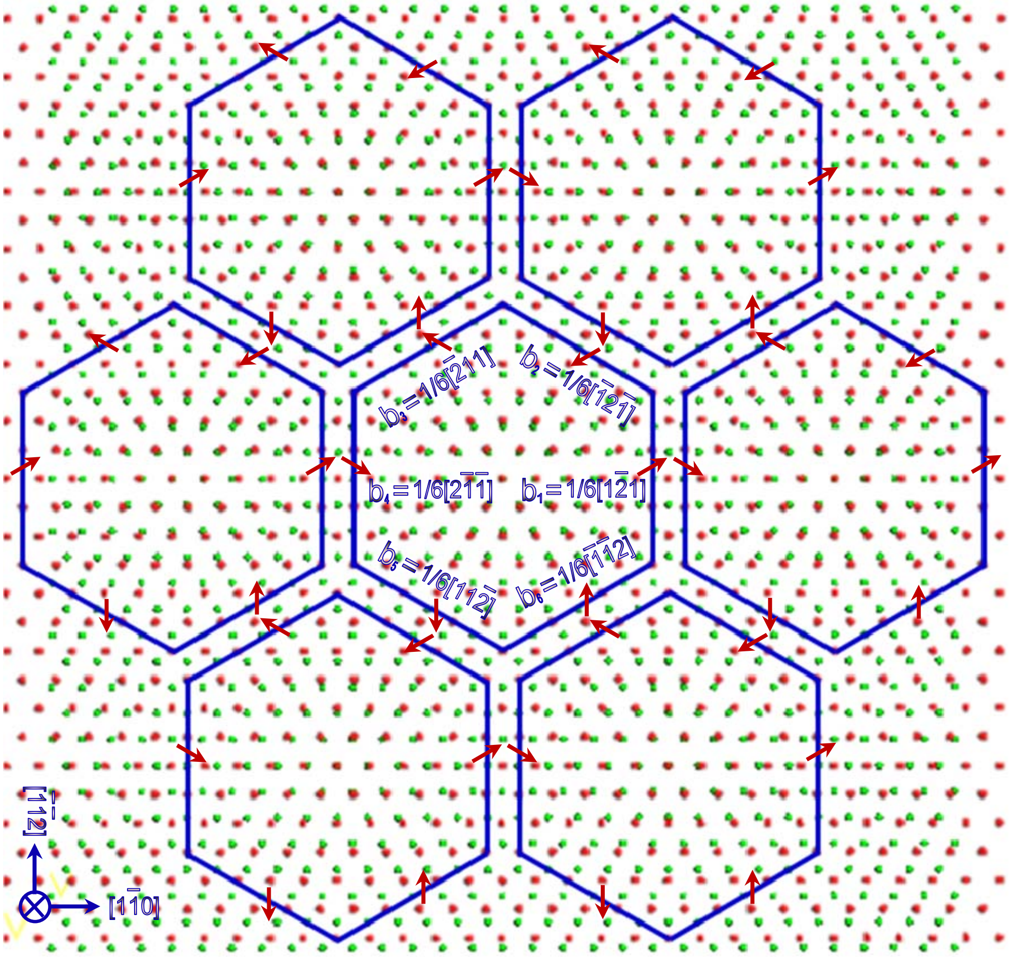

Supplementary Figure 2 | Model for describing the dissociation of misfit dislocations. A schematic diagram illustrating dissociation mechanism of misfit dislocations at the (111) diamond/c-BN interface. Each full edge dislocation dissociates into the two 60° 1/6<1 $\bar{2}$ 1> Shockley partial dislocations which are connected by a stacking fault.

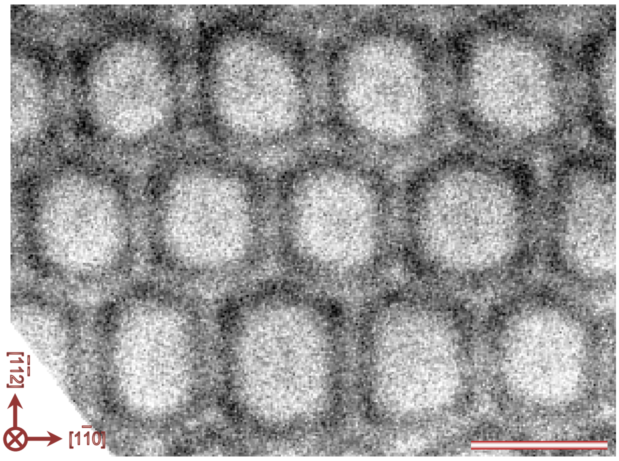

Supplementary Figure 3 | STEM imaging. A bright-field STEM image. The periodical hexagonal loops are composed of the Shockley partial misfit dislocations and continuous in-between stacking faults at the (111) diamond/c-BN interface. The electron beam is along the [111] zone axis. Scale bar, 20 nm.

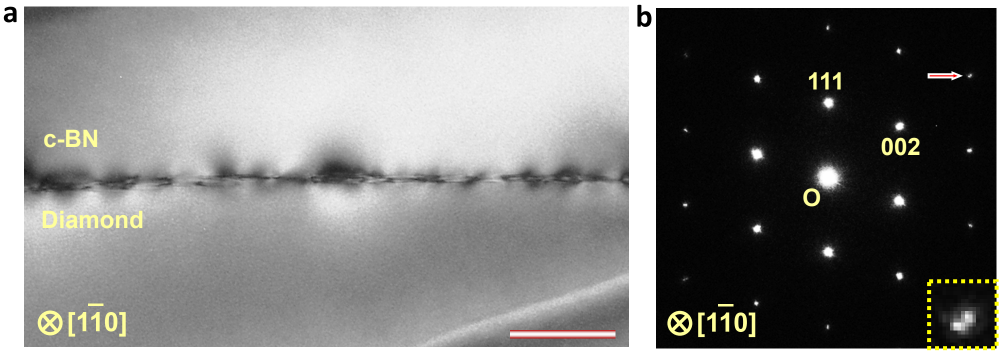

Supplementary Figure 4 | TEM image and SAED. A bright-field TEM image and the corresponding SAED pattern for the (111) diamond/c-BN interface taken along the [1$\bar{1}$0] zone axis. All misfit dislocations are inclined from this direction so that there is no periodic contrast of misfit dislocations. Scale bar, 50 nm.

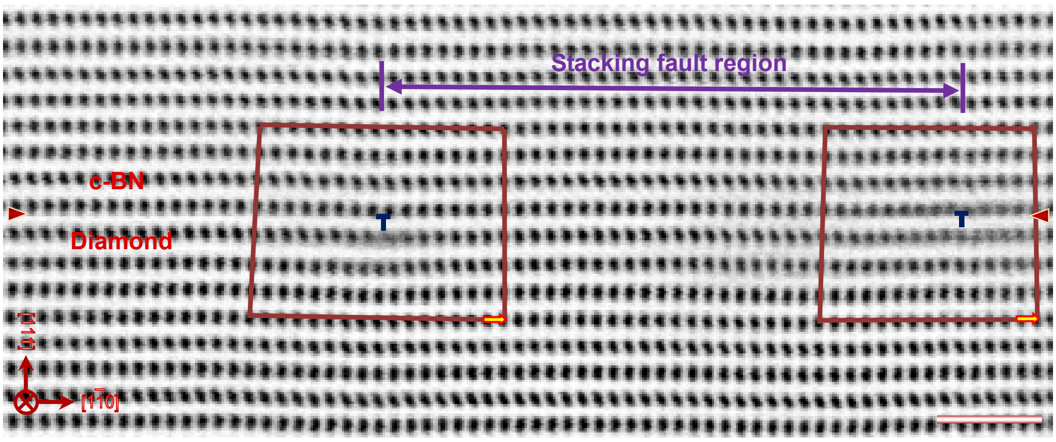

Supplementary Figure 5 | Annular bright-field imaging. Annular bright-field (ABF) STEM image along [11$\bar{2}$] direction. Two neighboring 60° 1/6<1$\bar{2}$1> Shockley partial dislocations are revealed. The interval region (~5 nm) between the two Shockley partial dislocations represents the stack fault. Scale bar, 1 nm.

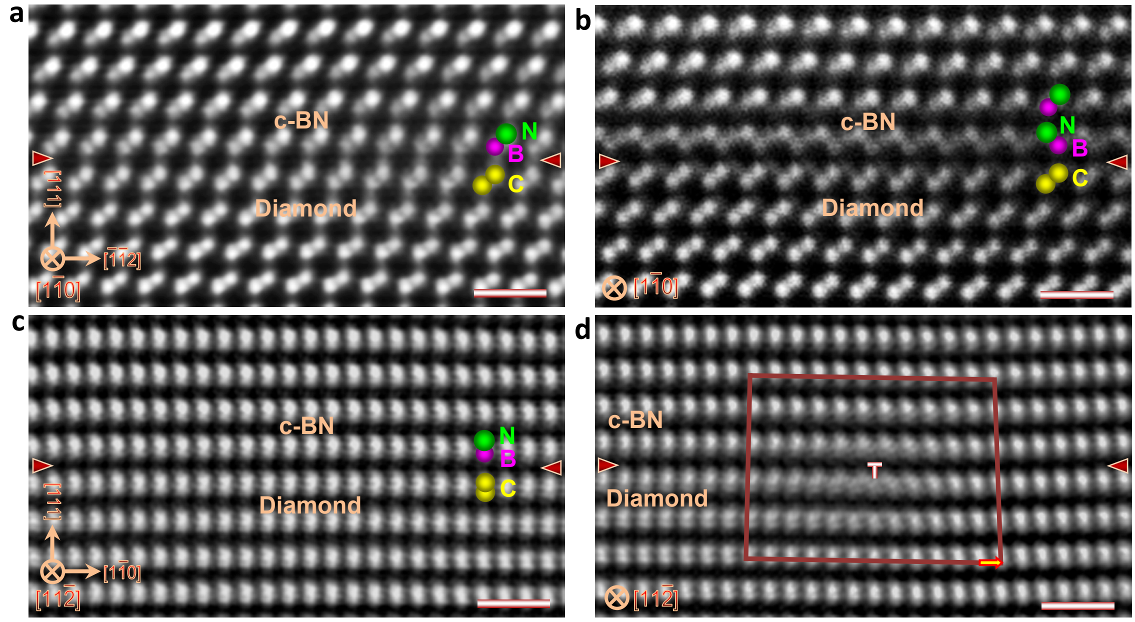

Supplementary Figure 6 | Atomic-scale structure of the heterointerface. a,b, HAADF STEM images along [1$\bar{1}$0] zone axis showing atomic structure of the directly-bonded coherent area (**a**) and stacking fault area (**b**). The interface is bonded by the B and C atoms. The stacking fault appears on c-BN side. **c,d,** HAADF STEM images along [11$\bar{2}$] zone axis showing atomic structures of the coherent region (**c**) and the area containing Shockley partial dislocations (**d**). The projected Burger vector is identified as 1/4[1$\bar{1}$0]. The interface is indicated by horizontal arrows. All images undergo low-pass filtering by the Fast Fourier Transform. Scale bar, 5 Å.

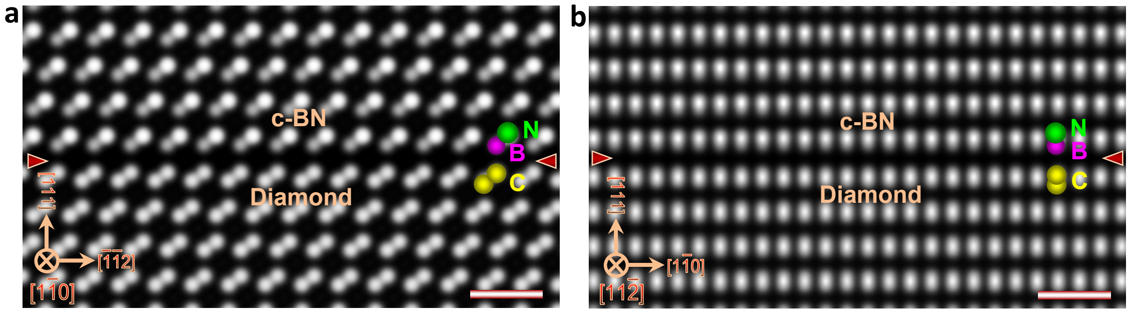

Supplementary Figure 7 | Simulated HAADF STEM images. a,b, Simulated HAADF STEM images along the [1$\bar{1}$0] (**a**) and [11$\bar{2}$] (**b**) zone axis. The simulated HAADF STEM images agree well with their corresponding experimental images. Scale bar, 5 Å.

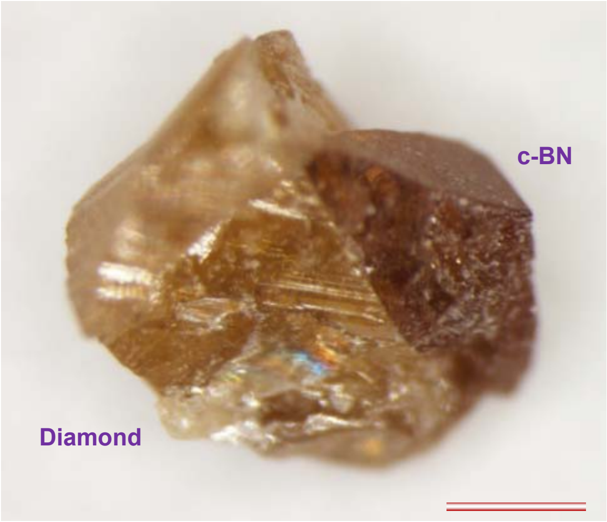

Supplementary Figure 8| A real picture of as-prepared diamond/c-BN heterojunction. The c-BN single crystal is grown on the surface of diamond seed crystal and has a size of ~0.5 mm. Scale bar, 0.5 mm.

Supplementary Discussion

Comparison of Energies

To examine whether the dissociation is energetically stable, we compared the energy of a 1/2<1$\bar{1}$0> perfect dislocation with the total energy of the dissociated two Shockley partial dislocations and the stacking fault. The elastic strain energy of a dislocation with a length of L can be expressed as follows:

$$E_{total} = \alpha G b^2 \cdot L, \quad (1)$$

where the α is a pre-factor ranging from 0.5 to 1, G is shear modulus, and b is length of Burgers vector. To estimate the energy of misfit dislocations, we adopted an average shear modulus of the diamond and c-BN, i.e. G = 438.4 GPa because the shear moduli of c-BN and diamond are 398.8 and 478 GPa, respectively[1,2]. The dislocation length is the length of hexagonal edge of dislocation loop and has a value of ~7.7 nm. The value of α is adopted as 0.75. The b has a value of 2.52 Å for the 1/2<1$\bar{1}$0> perfect dislocation and a value of 1.45 Å for the 1/6<112> Shockley partial dislocation. Based on Eq. 1, the strain energy of a 1/2<1$\bar{1}$0> perfect dislocation and a 1/6<112> Shockley partial dislocation is estimated to be 1.61×10^{-16} J and 5.32×10^{-17} J, respectively.

To estimate the stacking fault energy, we adopted an average stacking fault energy for the diamond (0.279 Jm^{-2}) and the c-BN (0.134 Jm^{-2})[3,4]. The stacking fault energy is hence estimated to be 0.207 Jm^{-2}. Here, the area of stacking fault equals multiplication of length of dislocation (i.e. 7.7 nm) and spacing of two dissociated partials (i.e. 5 nm). The area is thus estimated as 3.85×10^{-17} m^2. The stacking fault energy is therefore calculated to be 7.95×10^{-18} J.

According to the above calculations, the sum of the energy of two 1/6<112> Shockley partial dislocations and the connecting stacking fault is 1.14×10^{-16} J, which is smaller than the strain energy of a 1/2<1$\bar{1}$0> perfect dislocation (1.61×10^{-16} J). This indicates that

it is energetically favorable for the 1/2<1$\bar{1}$0> perfect dislocation to dissociate into two 1/6<112> Shockley partial dislocations and a stacking fault that connects the dislocations.

Supplementary References

1. Zhang, J. S., Bass, J. D., Taniguchi, T., Goncharov, A. F. Chang, Y. –Y. & Jacobsen, S. D. Elasticity of cubic boron nitride under ambient conditions. *J. Appl. Phys.* **109**, 063521 (2011)
2. Pierson, H. O. Handbook of Carbon, Graphite, Diamond and Fullerenes – Properties, Processing and Application. New Jersey, USA: Noyes Publications; 1993.
3. Nistor, L. C., Van Tendeloo, G. & Dinca, G. HRTEM studies of dislocations in cubic BN. *Phys. Stat. Sol. (a)* **201**, 2578–2582 (2004).
4. Pirouz, P., Cockayane, D. J. H., Sumida, N., Sir Hirsch, P. & Lang, A. R. Dissociation of dislocations in diamond. *Proc. R. Soc. Lond. A* **386,** 241–249 (1983).
